# Effects of hydroxychloroquine sulfate combined with aspirin and enoxaparin sodium therapy on perinatal outcomes in patients with undifferentiated connective tissue disease-related recurrent miscarriage: a retrospective clinical study

**DOI:** 10.1186/s12884-026-09337-1

**Published:** 2026-05-27

**Authors:** Liu-Cheng Pei, Ting Wang, Ya-Juan Guo, Qiu-Ling Yang, Wen-Di Liu, Cheng-Wei Jiang, Peng-Chao Yan, Xue-Jie Li, Chun-Jun Wang

**Affiliations:** 1 Department of Women’s Health, Wuhai Maternity and Child Healthcare Hospital, Wuhai, 016099 China; 2 School of Pharmacy, University College London, 29-39 Brunswick Square, London, WC1N 1AX UK

**Keywords:** Hydroxychloroquine sulphate, Aspirin, Enoxaparin sodium, Undifferentiated connective tissue disease, Repeated miscarriage

## Abstract

**Background:**

To evaluate the association between triple therapy (hydroxychloroquine sulfate, aspirin, and enoxaparin sodium) and pregnancy outcomes in patients with undifferentiated connective tissue disease (UCTD)-related recurrent miscarriage (RM).

**Methods:**

This retrospective cohort study included 135 pregnant women with recurrent miscarriage treated at a single center between January 2021 and August 2024. Patients were assigned to three groups according to treatment regimen: control group 1 (progesterone + enoxaparin sodium), control group 2 (aspirin + enoxaparin sodium), and the triple therapy group (hydroxychloroquine + aspirin + enoxaparin sodium). The primary outcome was live birth (≥ 28 weeks). Multivariable logistic regression analysis was performed to adjust for baseline differences using a parsimonious model including treatment group, maternal age, and gestational age at last pregnancy loss.

**Results:**

In unadjusted analysis, the triple therapy group had a significantly higher live birth rate (95.6%) than the control group 1 (80.0%) and control group 2 (77.8%) (*P* = 0.028). The higher live birth rate was mainly driven by increased fetal survival, despite a numerically higher preterm birth rate. After adjustment for baseline differences, the association between triple therapy and live birth was attenuated and did not reach statistical significance, although the direction of effect remained consistent with the unadjusted analysis. No significant difference was observed between control group 2 and control group 1 in the adjusted analysis. Gestational age at last pregnancy loss was significantly associated with live birth, whereas maternal age was not. No increase in adverse reactions or differences in neonatal outcomes was observed among the groups.

**Conclusion:**

Triple therapy with hydroxychloroquine, aspirin, and enoxaparin sodium was associated with improved live birth outcomes in patients with UCTD-related recurrent miscarriage. However, this association was attenuated and did not remain statistically significant after adjustment for baseline differences. Given the retrospective design and limited sample size, further prospective studies are required to confirm these findings.

**Supplementary Information:**

The online version contains supplementary material available at 10.1186/s12884-026-09337-1.

## Introduction

The age at childbearing among Chinese women has progressively increased in recent decades, largely driven by socioeconomic development, higher educational attainment, and evolving fertility policies such as the two- and three-child policies. Concurrently, fertility rates have declined markedly, primarily due to social and economic pressures, while the prevalence of infertility has been estimated at approximately 10–12% [1, 2]. The risk of miscarriage increases with maternal age and the number of miscarriages. The prevalence of recurrent miscarriage (RM) can be as high as 3% among women of reproductive age [3, 4]. The etiology of RM is highly complex and multifactorial. Immunological abnormalities and prothrombotic states, including antiphospholipid syndrome and thrombophilia, have been reported to account for a substantial proportion of cases, with some studies suggesting contributions of up to 50–60% [5–7]. Undifferentiated connective tissue disease (UCTD) predisposes to a prothrombotic state, and micro thrombosis can cause maternal placental dysfunction and adverse pregnancy outcomes [8–10].

Currently, there are no specific treatment protocols for UCTD-induced RM, and anticoagulants and immunotherapeutic drugs are commonly used for treatment. Enoxaparin sodium provides antithrombotic effects and modulates immune responses, improving endometrial blood supply and placental perfusion [11]. Aspirin is a commonly used antiplatelet agent in clinical practice, exerting anti-inflammatory, antipyretic, and analgesic effects through inhibition of platelet aggregation. However, low-dose aspirin monotherapy has not been shown to improve pregnancy outcomes or prevent recurrent miscarriage [12]. The combined use of aspirin and low-molecular-weight heparin (such as enoxaparin) has been reported to improve pregnancy outcomes, with live birth rates approaching approximately 70% in some clinical studies [13]. Conversely, in RM caused by UCTD, particularly refractory obstetric antiphospholipid syndrome, patients still experience pregnancy loss, preeclampsia or haemolysis, elevated liver enzymes, and low platelets syndrome, and preterm labor before 34 weeks of gestation [14]. Immunosuppressive therapy can be used for the management of UCTD. Hydroxychloroquine sulfate (hydroxychloroquine) possesses immunomodulatory properties and has been shown to reduce the binding of antiphospholipid antibodies to syncytiotrophoblasts and restore annexin A5 expression. In addition, it may alleviate inflammatory responses and improve trophoblast function, thereby contributing to improved placental development [15]. Adding hydroxychloroquine to aspirin combined with enoxaparin sodium in RM treatment has increased live birth rates; however, related studies in the current literature are limited [16]. Therefore, this retrospective study aims to analyze the effects of hydroxychloroquine combined with aspirin and enoxaparin sodium in patients with UCTD-related RM.

## Materials and methods

### General data

**Fig. 1:**
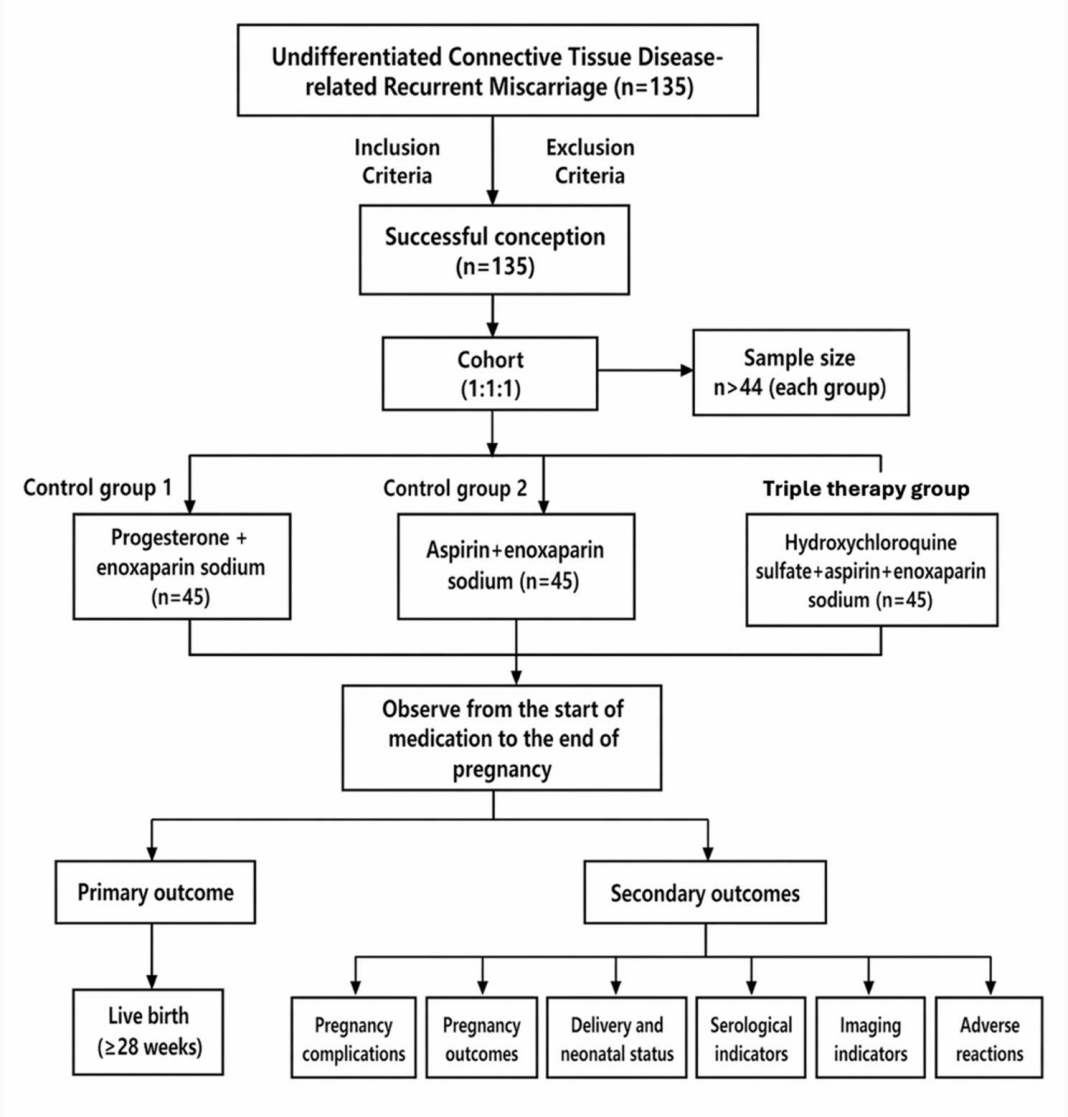
Flowchart

This retrospective cohort study included UCTD patients with a history of pregnancy complicated by RM who received treatment at Wuhai Maternity and Child Health Care Hospital between January 2021 and August 2024. A total of 135 patients were included. The selection of treatment methods was performed based on doctor-patient communication, and all participants provided written informed consent. This non-randomized treatment allocation may introduce selection bias, which was addressed by adjusting for baseline differences using multivariable regression analysis. We provided diagnosis and treatment options based on the patient’s condition during outpatient consultations, informed the patient of the advantages and disadvantages of different methods, and determined the treatment method through consultations with the patient. Patients were assigned to the following groups based on the treatment method adopted: control group 1 [progesterone (PRG) combined with enoxaparin sodium], control group 2 (aspirin combined with enoxaparin sodium), and the triple therapy group (hydroxychloroquine combined with aspirin and enoxaparin sodium). We enrolled 135 patients (45 patients per group) based on strict inclusion and exclusion criteria. All patients provided informed consent. This study was reviewed and approved by the Medical Ethics Committee of Wuhai Maternity and Child Health Care Hospital hospital (approval number: ZG202410) (Flowchart see Fig. 1). The sample size was determined based on available eligible cases during the study period.

### Inclusion and exclusion criteria

The inclusion criteria were as follows: (1) Age between 20 and 40 years, with normal fertility intention; (2) recurrent pregnancy loss defined as two or more consecutive unexplained spontaneous abortions with the same partner, excluding causes such as chromosomal abnormalities, uterine anatomical abnormalities, endocrine disorders, infection, and other clear non-immune-related factors. (3) UCTD diagnosis [17], with at least one symptom or sign suggesting a connective tissue disease (CTD) and at least one positive autoantibody, including antinuclear, anti-extractable nuclear antigen, anti-double-stranded DNA, antiphospholipid, and anti-cyclic citrullinated peptide antibodies, without meeting the classification criteria of a defined CTD. (4) Recurrent pregnancy loss [18], defined as two or more pregnancy losses of unknown origin in any trimester. (5) Voluntary agreement to participate in the corresponding treatment plan in this study, provide informed consent, and undergo regular follow-up and related examinations. (6) Complete medical records, including basic information, previous abortion history, UCTD diagnosis-related examination results, pre-treatment serum human chorionic gonadotropin (HCG), estradiol (E2), and PRG results, normal imaging and coagulation function indicators; treatment plan records and follow-up data during pregnancy are sufficient for research data extraction and analysis. (7) Provision of written informed consent.

The exclusion criteria were as follows: (1) Age < 20 or > 40 years, severe dysfunction of the heart, liver, kidney, or other vital organs, and inability to tolerate the study-related treatment drugs. (2) Recurrent pregnancy loss caused by non-immune-related factors, including chromosomal abnormalities (confirmed through chromosome testing of both spouses or aborted embryos), abnormal uterine anatomical structure (uterine mediastinum, intrauterine adhesion, uterine fibroids, etc.), endocrine diseases (uncontrolled hyperthyroidism or hypothyroidism, uncontrolled diabetes etc.), and reproductive tract infection (mycoplasma, chlamydia, toxoplasma, etc.). (3) Presence of other definite CTD types (systemic lupus erythematosus, rheumatoid arthritis, etc.), malignant tumors, and hematological diseases (thrombocytopenia, coagulation disorders, etc.). (4) Patients with bleeding disorders, active peptic ulcer, retinopathy, or allergy to study drugs (hydroxychloroquine, aspirin, enoxaparin sodium, and progestin), including contraindications to aspirin (active gastrointestinal ulcer and bleeding tendency) and enoxaparin sodium (severe coagulopathy and active bleeding). (5) Patients with severe infection or mental illness who are unable to cooperate with treatment. (6) Abnormal pregnancy, such as ectopic pregnancy, hydatidiform mole, fetal malformation, or use of immunosuppressant or anticoagulant drugs in the preceding 3 months. (7) Multiple and twin pregnancies. (8) Incomplete clinical records and missing critical information (such as treatment plan, follow-up records, and laboratory test results), which are insufficient for research data analysis, or patients lost to follow-up with unknown pregnancy outcome. (9) Infertility couples with other fertility factors. (10) Exclude retinal lesions. (11) Other causes of recurrent pregnancy loss disease: Hypothyroidism, Mullerian defects like septate uterus, balanced robertsonian parental translocations, intrauterine adhesions, chronic endometritis and perhaps even celiac disease, insulin resistance and smoking.

### Methods

In the control group 1 (PRG combined with enoxaparin sodium group), enoxaparin sodium (0.6 mL/syringe, 6000 AXa IU, Drug Approval Number: H20223859) was added based on PRG treatment, with the PRG dosage remaining unchanged. Enoxaparin sodium was initiated after ovulation, administered subcutaneously at a prophylactic dose of 100 u/kg body weight/day. Upon pregnancy confirmation, the dose was adjusted to a therapeutic level of 200 u/kg body weight/day through subcutaneous injection. The drug was discontinued if laboratory serological antibody test results were negative for three consecutive examinations at one-month intervals after pregnancy.

In the control group 2 (aspirin combined with enoxaparin sodium group), aspirin was initiated at 3 months before pregnancy. Bayer aspirin (Bayer HealthCare; 100 mg/tablet/day; Drug Approval Number: H20171021) was administered orally in patients with body weight ≥ 50 kg, and domestically-produced aspirin (Nanjing Baijingyu Pharmaceutical Co., Ltd; 25 mg/tablet; Drug Approval Number: H32026500) was administered orally at 75 mg/day in patients with body weight < 50 kg. The drug was discontinued if laboratory serological antibody test results were negative for three consecutive examinations at one-month intervals after pregnancy.

In the triple therapy group (hydroxychloroquine combined with aspirin and enoxaparin sodium group), hydroxychloroquine [Plaquenil (0.2 g/tablet) or Fenle (0.1 g/tablet); SPH Zhongxi Pharmaceutical Co., Ltd.; Drug Approval Number: H19990264] was added based on PRG, aspirin, and enoxaparin sodium treatment. Hydroxychloroquine was initiated at 3 months before pregnancy at a usual dose of 200 mg/day through oral administration. For patients positive for anti-sjögren’s syndrome A antibodies (SSA), anti-sjögren’s syndrome B antibodies (SSB), and double-stranded DNA antibodies, the usual dose of 400 mg/day was administered orally. For pregnant women who were overweight, a dose of < 6.75 mg/kg body weight per day was adopted, with the total daily amount capped at 400 mg. The drug was discontinued if laboratory serological antibody test results were negative for three consecutive examinations at 1-month intervals after pregnancy. (Note: a fundoscopic examination was required before administration of hydroxychloroquine, and a re-examination was required after > 6 months of drug use).

### Observational indicators

The primary outcome of this study was live birth, defined as delivery at ≥ 28 weeks of gestation.

Secondary outcomes included:

1. Pregnancy complications: These include gestational diabetes mellitus (diagnosed using a 75-gram oral glucose tolerance test at 24–28 weeks of gestation if fasting blood sugar, 1-hour post-load, or 2-hour post-load level was ≥5.1 mmol/L, ≥10.0 mmol/L, or ≥8.5 mmol/L, respectively), gestational hypertension (defined as blood pressure ≥140/90 mmHg after 20 weeks of gestation, resolving within 12 weeks after delivery), premature membrane rupture (natural prenatal rupture of the membranes), placental abruption (detachment of the placenta from its normal position on the uterine wall after 20 weeks of gestation and before delivery), polyhydramnios (B-mode ultrasound indicating amniotic fluid volume [AFV] ≥8 cm and amniotic fluid index [AFI] ≥25 cm), oligohydramnios (B-mode ultrasound indicating AFV ≤2 cm and AFI ≤5 cm), fetal growth restriction (fetal weight <10th percentile for its gestational age), postpartum hemorrhage (≥500 mL blood loss for vaginal delivery and ≥1000 mL blood loss for caesarean section within 24 h after delivery).
2. Pregnancy outcomes: These include miscarriage (termination at <28 weeks of gestation, with fetal weight <1000 g), stillbirth (intrauterine fetal death after 20 weeks of gestation), preterm delivery (delivery at ≥28 and <37 weeks of gestation), and full-term delivery (delivery at ≥37 and <42 weeks of gestation).
3. Delivery and neonatal status: vaginal delivery rate, caesarean section rate, and neonatal status (survival and asphyxia).
4. Serological indicators: These include HCG, E2, PRG, blood biochemical indicators, routine blood test indicators, and coagulation indices (D-dimer, activated partial thromboplastin time, prothrombin time, thrombin time, international normalized ratio, and the four new coagulation indices, namely thrombomodulin, thrombin-antithrombin complex, plasmin-α2-plasmininhibitor complex, and tissue plasminogen activator-inhibitor complex).
5. Imaging indicators: Gestational sac size, fetal pole length, gestational sac–embryo size discrepancy, and uterine artery blood flow (assessed using vaginal ultrasound before and after treatment) were compared. HCG, E2, and PRG levels were compared with vaginal ultrasound findings obtained on the same day, and longitudinal changes in these parameters across different time points were further analyzed to comprehensively evaluate embryonic development.
6. Adverse reactions: Patients were observed for adverse reactions, such as gastrointestinal reactions, abnormal liver function, bleeding tendency, rash, thrombocytopenia, and blurred vision.

### Statistical methods

All statistical analyses were performed using SPSS version 26.0 (IBM Corp., Armonk, NY, USA).

Continuous variables were first assessed for normality using the Shapiro–Wilk test and were considered approximately normally distributed. Normally distributed data are presented as mean ± standard deviation (SD) and were compared using one-way analysis of variance (ANOVA). Homogeneity of variances was evaluated using Levene’s test. If the assumption of equal variances was violated, Welch’s ANOVA was applied.

For non-normally distributed data, results are presented as median and interquartile range (IQR), and comparisons were performed using the Kruskal–Wallis test.

Categorical variables are presented as frequencies and percentages and were compared using the chi-square test or Fisher’s exact test, as appropriate.

To account for baseline imbalances and potential confounding factors, multivariable logistic regression analysis was performed to evaluate the association between treatment group and live birth outcome (≥ 28 weeks of gestation). Variables included in the adjusted model were treatment group, maternal age, and gestational age at last pregnancy termination, selected based on clinical relevance and baseline differences.

Two models were constructed: Model 1 (unadjusted), including treatment group only, and Model 2 (adjusted), including treatment group and potential confounders. Results are presented as odds ratios (OR) with 95% confidence intervals (CI).

A two-sided P value < 0.05 was considered statistically significant.

This study was reported in accordance with the STROBE guidelines (Supplemental Table 1). Missing data were minimal and handled using complete-case analysis.

## Results

### Baseline characteristics

A total of 135 patients were included in this study, with 45 patients in each group. Baseline characteristics are presented in Table 1. Significant differences were observed in maternal age, gravidity, and gestational age at last pregnancy termination among the groups (*P* < 0.05). Patients in the triple therapy group were older and had earlier gestational age at last pregnancy termination compared with the control groups. No significant differences were observed among the groups in BMI, parity, or number of previous miscarriages (*P* > 0.05).

**Table 1:** Baseline characteristics of patients in each group (*Mean* ± *SD*)

Group	Age (years)	BMI (kg/m2)	Gravidity	Parity	Number of miscarriages	Gestational age at last termination of pregnancy (weeks)
Control group 1 (*n* = 45)	28.5 ± 3.2	22.4 ± 3.7	3.2 ± 0.9	0.3 ± 0.6	2.9 ± 0.8	8.0 ± 1.5
Control group 2 (*n* = 45)	31.4 ± 4.5	21.9 ± 2.5	3.4 ± 1.0	0.2 ± 0.4	3.3 ± 0.9	8.7 ± 4.9
Triple therapy group (*n* = 45)	30.8 ± 3.4	22.0 ± 2.4	2.9 ± 0.9	0.2 ± 0.5	2.9 ± 0.8	7.5 ± 0.8
F	7.718	0.483	3.892	0.708	2.950	8.184
P	0.001*	0.618	0.023#	0.495	0.056	< 0.001^*#^

Statistical tests were selected based on data distribution and are described in the Methods section. *: *P* < 0.05 compared with control group 1, ^#^: *P* < 0.05 compared with control group 2

Control group 1: progesterone combined with enoxaparin sodium; control group 2: aspirin combined with enoxaparin sodium; Exposed group: hydroxychloroquine sulphate combined with aspirin and enoxaparin sodium

### Primary outcomes: live birth

In the unadjusted analysis, the live birth rate was significantly higher in the triple therapy group (95.6%) compared with control group 1 (80.0%) and control group 2 (77.8%) (*P* = 0.028) (Table 2). This improvement in live birth rate should be interpreted with caution, as it was accompanied by a numerically higher preterm birth rate in the triple therapy group. To account for baseline differences among groups, multivariable logistic regression analysis was performed using a parsimonious model including treatment group, maternal age, and gestational age at last pregnancy loss (Table 3).

**Table 2:** Comparison of pregnancy outcomes among groups (*n* [%])

Group(%)	Miscarriage(Gestational age)	Preterm birth	Full-term birth
Control group 1 (*n* = 45)	9(20.0) 9.33 ± 1.9	1(2.2)	35(77.8)
Control group 2 (*n* = 45)	10(22.2) 8.7 ± 1.8	0(0)	35(77.8)
Triple therapy group (*n* = 45)	2(4.4) 9.0 ± 0.0	5(11.1)	38(84.4)
Fisher’s exact test	10.857		
P	0.028*		

Statistical tests were selected based on data distribution and are described in the Methods section. *: *P* < 0.05 compared with control group 1, ^#^: *P* < 0.05 compared with control group 2

Control group 1: progesterone combined with enoxaparin sodium; Control group 2: aspirin combined with enoxaparin sodium; Triple therapy group: hydroxychloroquine combined with aspirin and enoxaparin sodium

**Table 3:** Multivariable logistic regression analysis of live birth

Variable	OR	95% CI	*P* value
Group (Control 2 vs. Control 1)	1.01	0.27–3.80	0.988
Group (Triple therapy group vs. Control 1)	3.15	0.54–18.49	0.203
Age	0.94	0.82–1.07	0.349
Gestational Age at Last Loss	0.34	0.17–0.66	0.002

*Abbreviations: *OR* Odds ratio, *CI* Confidence interval. Control group 1 was used as the reference category. The model was adjusted for age, and gestational age at last loss

In the adjusted model, gestational age at last pregnancy loss was significantly associated with live birth (OR = 0.34, 95% CI: 0.17–0.66, *P* = 0.002). The association between treatment group and live birth was attenuated after adjustment and did not reach statistical significance (overall *P* = 0.371). Compared with control group 1, the triple therapy group showed a higher likelihood of live birth (OR = 3.15, 95% CI: 0.54–18.49, *P* = 0.203), although this difference was not statistically significant. Maternal age was not significantly associated with live birth in the adjusted model (*P* = 0.349).

The attenuation of the treatment effect after adjustment may be explained by the limited sample size and the small number of miscarriage events, which reduced statistical power.

### Secondary outcomes: pregnancy complications

The incidence of gestational diabetes mellitus in the triple therapy group (2.2%; 1 out of 45) was significantly lower than that in the control group 2 (15.6%; 7 out of 45; *χ²*=6.696; *P* = 0.035), whereas no significant differences were observed between the triple therapy group and control group 1 (4.4%; 2 out of 45; *P* > 0.05).

Furthermore, the incidence of term premature rupture of membranes in the triple therapy group (26.67%; 12 out of 45) was significantly higher than that in control group 2 (6.5%; 3 out of 45; *χ²*=6.625; *P* = 0.036), with no significant differences between the triple therapy group and control group 1 (15.6%; 7 out of 45; *P* > 0.05).

The incidence of other complications (gestational hypertension, placental abruption, postpartum hemorrhage) did not significantly differ among the groups (all *P* > 0.05). Additionally, the overall incidence of pregnancy complications did not significantly differ among the groups (χ²=3.253; *P* = 0.197; Table 4). Although individual complications differed, the composite outcome did not reach statistical significance, possibly due to the limited sample size.

**Table 4:** Comparison of pregnancy complications among groups (*n* [%])

Group (%)	Gestational diabetes mellitus	Gestational hypertension	Term premature rupture of membranes	Placental abruption	Postpartum haemorrhage	Total incidence
Control group 1 (*n* = 45)	2 (4.4)	7 (15.6)	7 (15.6)	0 (0.0)	0 (0.0)	16 (35.6)
Control group 2 (*n* = 45)	7 (15.6)	4 (8.3)	3 (6.5)	0 (0.0)	0 (0.0)	14 (31.1)
Triple therapy group (*n* = 45)	1 (2.2)	7 (15.6)	12 (26.7)	2 (4.4)	0 (0.00)	22 (48.9)
χ^2^	6.696	1.154	6.625	4.060	-	3.253
P	0.035^#^	0.562	0.036^*#^	0.131	-	0.197

^*^: *P* < 0.05 compared with control group 1, ^#^: *P* < 0.05 compared with control group 2

Control group 1: progesterone combined with enoxaparin sodium; Control group 2: aspirin combined with enoxaparin sodium; Triple therapy group: hydroxychloroquine combined with aspirin and enoxaparin sodium

### Secondary outcomes: delivery and neonatal outcomes

The caesarean section rate in the triple therapy group did not significantly differ from that of control groups 1 and 2 (*P* > 0.05). Differences in Apgar score and birth weight were not statistically significant among the groups (*P* > 0.05) (Supplemental Table 2).

### Secondary outcomes: laboratory and imaging parameters

The post-/pre-treatment HCG, E2, and PRG ratios of the triple therapy group did not differ significantly from those of control groups 1 and 2 (*P* > 0.05). However, serological indicator levels at 2 weeks post-treatment were significantly increased compared with the pre-treatment values across the groups, with greater increases in the triple therapy group (Table 5).

**Table 5:** Comparison of post-treatment/pre-treatment ratios of serological indicators among groups (*Mean* ± *SD* and *n* [%])

Group	HCG (mIU)	E2 (pg)	PRG (ng)
	2 weeks post-treatment/pre-treatment ratio	2 weeks post-treatment/pre-treatment ratio	2 weeks post-treatment/pre-treatment ratio
Control group 1 (*n* = 45)	2.9 ± 1.5	2.3 ± 1.2	1.3 ± 0.5
Control group 2 (*n* = 45)	4.6 ± 5.4	2.5 ± 2.6	1.2 ± 0.4
Triple therapy group (*n* = 45)	3.8 ± 4.3	1.9 ± 0.9	1.3 ± 0.5
F	2.509	1.472	1.123
P	0.085	0.233	0.329

Statistical tests were selected based on data distribution and are described in the Methods section. *: *P* < 0.05 compared with control group 1, ^#^: *P* < 0.05 compared with control group 2

Control group 1: progesterone combined with enoxaparin sodium; Control group 2: aspirin combined with enoxaparin sodium; Triple therapy group: hydroxychloroquine combined with aspirin and enoxaparin sodium; HCG Human chorionic gonadotropin, E2 Estradiol, PRG Progesterone

Among the post-/pre-treatment ratios of imaging indicators, only the fetal pole length ratio showed a statistically significant difference among the three groups (F = 6.887; *P* < 0.001). The ratio in the triple therapy group (4.96 ± 2.28) was significantly lower than that in control group 2 (5.31 ± 2.80; ^#^*P* < 0.05), with no significant difference observed between the triple therapy group and control group 1 (4.35 ± 2.19; *P > 0.05). No significant group differences were observed for the other three indicators (all *P* > 0.05). Ratios of the gestational sac size were 1.25 ± 0.35 (control group 1), 1.28 ± 0.22 (control group 2), and 1.26 ± 0.22 (triple therapy group) (F = 0.109; *P* = 0.897). Gestational sac-embryo size discrepancy was 1.32 ± 1.13 (control group 1), 1.25 ± 0.47 (control group 2), and 1.18 ± 0.59 (triple therapy group) (F = 0.334; *P* = 0.717). Additionally, ratios of uterine artery blood flow were 0.75 ± 0.36 (control group 1), 0.83 ± 0.49 (control group 2), and 0.78 ± 0.25 (triple therapy group) (F = 0.429; *P* = 0.652) (Supplemental Table 3).

### Secondary outcomes: adverse reactions

Comparison of the incidence rates of gastrointestinal reactions, bleeding tendency, rash, thrombocytopenia, and blurred vision revealed no significant differences in adverse reactions among the groups (*P* > 0.05). The proportion of liver function abnormalities, PRG reactions, vaginal bleeding, and rash occurrence was slightly higher; however, the difference was not statistically significant. The triple therapy group experienced no additional adverse reactions (Supplemental Table 4).

## Discussion and limitations

The observed association may be biologically plausible given the known roles of immune dysregulation, thrombosis, and placental dysfunction in UCTD-related recurrent miscarriage. In the present study, triple therapy was associated with a higher live birth rate compared with dual therapy. However, given the retrospective and observational nature of this study, causal relationships cannot be established. The underlying mechanisms of the observed associations were not directly investigated in this study and therefore should be interpreted with caution. Potential mechanisms have been proposed in previous studies, although they were not directly evaluated in this study.

Importantly, after adjusting for baseline differences and potential confounders, the association between triple therapy and live birth was attenuated and did not reach statistical significance, suggesting that the observed benefit may be partly influenced by baseline differences and limited statistical power. Gestational age at last pregnancy loss was identified as a significant predictor of live birth, whereas maternal age was not significantly associated with the outcome in the adjusted model. The relatively wide confidence intervals observed for the treatment effect indicate some uncertainty, which is likely related to the limited sample size and the small number of miscarriage events. These findings highlight the important role of disease severity and placental function in UCTD-related recurrent miscarriage. In line with this, recent research has identified novel molecular markers associated with pregnancy outcomes. For example, a recent study reported that CYR61, a protein involved in placental development and angiogenesis, may serve as a potential biomarker for recurrent miscarriage [19]. This evidence further supports the role of placental dysfunction and immune imbalance in the pathogenesis of recurrent miscarriage.

The incidence of gestational diabetes mellitus was lower in the triple therapy group compared with control group 2, although no difference was observed compared with control group 1. These findings should be interpreted cautiously due to the limited sample size.

Triple therapy may exert complementary effects through immunomodulation, antithrombotic activity, and support of placental function. Hydroxychloroquine may regulate immune responses [15, 20, 21], aspirin may inhibit platelet aggregation and improve uteroplacental perfusion, and enoxaparin may reduce hypercoagulability while supporting trophoblast-related processes [21, 22]. However, these mechanisms were not directly evaluated in this study and should be interpreted as biologically plausible rather than confirmed.

The triple therapy group showed more favorable unadjusted pregnancy outcomes despite having more severe baseline characteristics. However, after adjustment for baseline differences, the association with live birth was attenuated and did not reach statistical significance, suggesting potential confounding and limited statistical power.

Patients in the triple therapy group were older and had an earlier gestational age at last pregnancy termination, suggesting a potentially more severe baseline condition. Despite these differences, the triple therapy group demonstrated more favorable unadjusted pregnancy outcomes compared with the dual therapy groups. However, after adjustment for baseline differences, the association between treatment group and live birth was attenuated and did not reach statistical significance. Therefore, these findings should be interpreted as suggesting a potential benefit of hydroxychloroquine combined with aspirin and enoxaparin sodium, rather than confirming treatment effectiveness. A three-arm, multicenter, open-label randomized controlled trial (the Immunosuppressant Regimens for Living Fetuses trial) has investigated the use of hydroxychloroquine and low-dose prednisone for recurrent pregnancy loss in women with UCTD; however, the final results are not yet available [23]. In this context, our study provides real-world evidence that may help inform treatment selection for UCTD-related recurrent miscarriage, although further prospective studies are required. The mechanisms underlying these observations remain unclear and were not directly investigated in the present study. Although changes in serological and imaging parameters were observed, their clinical significance and underlying mechanisms remain uncertain. The observed improvements in serological and imaging indicators may also reflect enhanced embryonic developmental potential, although causal mechanisms cannot be established.

Safety analysis indicated no significant differences in the overall incidence of adverse reactions among groups. The triple therapy group did not exhibit additional adverse events and primarily experienced mild gastrointestinal symptoms and mild liver function abnormalities, with no cases of severe hemorrhage, rash, thrombocytopenia, or visual impairment. These findings suggest that the triple therapy regimen was generally well tolerated in this cohort.

This study has some limitations. First, its retrospective, non-randomized design introduces strong selection bias, and although multivariable regression was used to adjust for baseline differences, residual confounding cannot be excluded. However, as a real-world study, it better reflects clinical practice. Second, the single-center sample (only from Wuhai Maternity and Child Health Care Hospital) limits the generalizability of the results to other populations. Third, the study only focused on perinatal outcomes, lacking long-term follow-up of maternal UCTD recurrence and neonatal growth. Fourth, it did not quantify the correlation between autoantibody levels and pregnancy outcomes, and mechanistic discussions relied on existing literature rather than direct experimental data from this study. Further investigations are required to support the broader application of this treatment algorithm.

## Conclusions

In this retrospective study, triple therapy comprising hydroxychloroquine, aspirin, and enoxaparin sodium was associated with enhanced live birth outcomes in individuals with UCTD-related recurrent miscarriage. However, this association was attenuated and did not remain statistically significant after adjustment for baseline differences. Besides the main outcome, triple therapy was associated with positive changes in other pregnancy-related factors, such as a lower rate of gestational diabetes mellitus and improvement in hormonal and imaging parameters. Additionally, there was no increase in adverse events or decline in neonatal outcomes, thereby supporting the overall safety profile of this regimen. However, due to the retrospective approach and the relatively small sample size, these findings must be regarded with caution. Additional extensive prospective investigations are necessary to validate these findings and to elucidate the therapeutic significance of triple therapy in UCTD-associated recurrent miscarriage.

## Supplementary Information

Supplementary Material 1.

## Data Availability

The data generated in the present study may be requested from the corresponding author.

## Abbreviations

AFI: Amniotic fluid index
AFV: Amniotic fluid volume
BMI: Body mass index
NETs: Neutrophil extracellular traps
RM: Recurrent miscarriage
HCG: serum human chorionic gonadotropin
UCTD: Undifferentiated connective tissue disease
PRG: Progesterone
E2: Estradiol
