# Supplementary material for: Effects of hydroxychloroquine sulfate combined with aspirin and enoxaparin sodium therapy on perinatal outcomes in patients with undifferentiated connective tissue disease-related recurrent miscarriage: a retrospective clinical study
Source: BMC Pregnancy Childbirth. 2026 May 27;26:801. doi: 10.1186/s12884-026-09337-1 (PMC13393678; doi:10.1186/s12884-026-09337-1)
Supplement: Supplementary file 1 — Supplementary Material 1. [file 12884_2026_9337_MOESM1_ESM.docx]

**Effects of Hydroxychloroquine Sulfate Combined with Aspirin and Enoxaparin Sodium Therapy on Perinatal Outcomes in Patients with Undifferentiated Connective Tissue Disease-related Recurrent Miscarriage: A Retrospective Clinical Study**

**Liu-Cheng Pei**^1^**, Ting Wang**^1*^**, Ya-Juan Guo**^1^**, Qiu-Ling Yang**^1^**, Wen-Di Liu**^1^**, Cheng-Wei Jiang**^2^**, Peng-Chao Yan**^1^**, Xue-Jie Li**^1^**, Chun-Jun Wang**^1*^

1 Department of Women’s Health, Wuhai Maternity and Child Healthcare Hospital, 016099, Wuhai, China

2 School of Pharmacy, University College London, 29-39 Brunswick Square, London WC1N 1AX, UK

*Co-Correspondence: Ting Wang and Chun-Jun Wang contributed equally

Tel: 010-13947347665

Email：Ting Wang Email: 574707256@qq.com；Chun-Jun Wang Email: [whsfywcj@163.com](mailto:whsfywcj@163.com,).

**Contents**

[Supplemental Table 1. STROBE Statement 2](#_Toc228243915)

[Supplemental Table 2. Comparison of vaginal delivery rate, caesarean section rate, and neonatal status of surviving foetuses among groups (x±s, n [%]) 4](#_Toc228243916)

[Supplemental Table 3. Comparison of post-/pre-treatment ratios of imaging indicators among groups (x±s) 4](#_Toc228243917)

[Supplemental Table 4. Comparison of adverse reactions among groups (n [%]) 5](#_Toc228243918)

Supplemental Table 1. STROBE Statement

|  | **Item No** | **Recommendation** | **Page No.** |
| --- | --- | --- | --- |
| **Title and abstract** | 1 | (*a*) Indicate the study’s design with a commonly used term in the title or the abstract | 0-1 |
|  |  | (*b*) Provide in the abstract an informative and balanced summary of what was done and what was found | 1 |
| Introduction | | |  |
| Background/rationale | 2 | Explain the scientific background and rationale for the investigation being reported | 2-3 |
| Objectives | 3 | State specific objectives, including any prespecified hypotheses | 3 |
| Methods | | |  |
| Study design | 4 | Present key elements of study design early in the paper | 4 |
| Setting | 5 | Describe the setting, locations, and relevant dates, including periods of recruitment, exposure, follow-up, and data collection | 4 |
| Participants | 6 | (*a*) Give the eligibility criteria, and the sources and methods of selection of participants. Describe methods of follow-up | 4-5 |
|  |  | (*b*) For matched studies, give matching criteria and number of exposed and unexposed | N/A |
| Variables | 7 | Clearly define all outcomes, exposures, predictors, potential confounders, and effect modifiers. Give diagnostic criteria, if applicable | 6-7 |
| Data sources/ measurement | 8 | For each variable of interest, give sources of data and details of methods of assessment (measurement). Describe comparability of assessment methods if there is more than one group | 6-7 |
| Bias | 9 | Describe any efforts to address potential sources of bias | 4 + 8 |
| Study size | 10 | Explain how the study size was arrived at | 4 |
| Quantitative variables | 11 | Explain how quantitative variables were handled in the analyses. If applicable, describe which groupings were chosen and why | 7-8 |
| Statistical methods | 12 | (*a*) Describe all statistical methods, including those used to control for confounding | 8 |
|  |  | (*b*) Describe any methods used to examine subgroups and interactions | N/A |
|  |  | (*c*) Explain how missing data were addressed | 5 |
|  |  | (*d*) If applicable, explain how loss to follow-up was addressed | 5 |
|  |  | (*e*) Describe any sensitivity analyses | N/A |
| Results | | |  |
| Participants | 13 | (a) Report numbers of individuals at each stage of study—eg numbers potentially eligible, examined for eligibility, confirmed eligible, included in the study, completing follow-up, and analysed | 6 |
|  |  | (b) Give reasons for non-participation at each stage | 5 |
|  |  | (c) Consider use of a flow diagram | 6 |
| Descriptive data | 14 | (a) Give characteristics of study participants (eg demographic, clinical, social) and information on exposures and potential confounders | 9-10 |
|  |  | (b) Indicate number of participants with missing data for each variable of interest | N/A |
|  |  | (c) Summarise follow-up time (eg, average and total amount) | N/A |
| Outcome data | 15 | Report numbers of outcome events or summary measures over time | 10-12 |
| Main results | 16 | (*a*) Give unadjusted estimates and, if applicable, confounder-adjusted estimates and their precision (eg, 95% confidence interval). Make clear which confounders were adjusted for and why they were included | 10-11 |
|  |  | (*b*) Report category boundaries when continuous variables were categorized | N/A |
|  |  | (*c*) If relevant, consider translating estimates of relative risk into absolute risk for a meaningful time period | N/A |
| Other analyses | 17 | Report other analyses done—eg analyses of subgroups and interactions, and sensitivity analyses | 11-13 |
| Discussion | | |  |
| Key results | 18 | Summarise key results with reference to study objectives | 14 |
| Limitations | 19 | Discuss limitations of the study, taking into account sources of potential bias or imprecision. Discuss both direction and magnitude of any potential bias | 18 |
| Interpretation | 20 | Give a cautious overall interpretation of results considering objectives, limitations, multiplicity of analyses, results from similar studies, and other relevant evidence | 14-18 |
| Generalisability | 21 | Discuss the generalisability (external validity) of the study results | 18 |
| Other information | | |  |
| Funding | 22 | Give the source of funding and the role of the funders for the present study and, if applicable, for the original study on which the present article is based | 19 |

Supplemental Table 2. Comparison of vaginal delivery rate, caesarean section rate, and neonatal status of surviving foetuses among groups (x±s, n [%])

| Group（%） | Vaginal delivery rate | Caesarean section rate | Neonatal status | |
| --- | --- | --- | --- | --- |
|  |  |  | Apgar score (points) | Birth weight (kg) |
| Control group 1 (*n*=36 of 45) | 15 (41.7) | 21 (58.3) | 9.7±0.33 | 3.2±0.6 |
| Control group 2 (*n*=35 of 45) | 11 (31.4) | 24 (68.6) | 9.7±0.30 | 3.3±0.4 |
| Exposed group (*n*=43 of 45) | 16 (37.2) | 27 (62.8) | 9.8±0.15 | 3.0±0.5 |
| χ^2^/F | 0.803 | | 0.16 | 0.7 |
| P | 0.669 | | 0.852 | 0.499 |

Note: ^*^: *P*<0.05 compared with control group 1, ^#^: *P*<0.05 compared with control group 2.

Control group 1: progesterone combined with enoxaparin sodium; control group 2: aspirin combined with enoxaparin sodium; exposed group: hydroxychloroquine sulphate combined with aspirin and enoxaparin sodium.

Supplemental Table 3. Comparison of post-/pre-treatment ratios of imaging indicators among groups (*x*±*s*)

| Group（%） | Foetal pole length (mm) | Gestational sac size  (mm) | Gestational sac-embryo size discrepancy (mm) | Uterine artery blood flow |
| --- | --- | --- | --- | --- |
|  | 2 weeks post-treatment/pre-treatment ratio | 2 weeks post-treatment/pre-treatment ratio | 2 weeks post-treatment/pre-treatment ratio | 2 weeks post-treatment/pre-treatment ratio |
| Control group 1 (*n*=45) | 4.4±2.2 | 1.3±0.4 | 1.3±1.1 | 0.8±0.4 |
| Control group 2 (*n*=45) | 5.3±2.8 | 1.3±0.2 | 1.3±0.5 | 0.8±0.5 |
| Triple therapy group (*n*=45) | 5.0±2.3 | 1.3±0.2 | 1.2±0.6 | 0.8±0.3 |
| F | 6.887 | 0.109 | 0.334 | 0.429 |
| P | <0.001^#^ | 0.897 | 0.717 | 0.652 |

Note: ^*^: *P*<0.05 compared with control group 1, ^#^: *P*<0.05 compared with control group 2.

Control group 1: progesterone combined with enoxaparin sodium; control group 2: aspirin combined with enoxaparin sodium; exposed group: hydroxychloroquine sulphate combined with aspirin and enoxaparin sodium.

Supplemental Table 4. Comparison of adverse reactions among groups (*n* [%])

| Group（%） | Gastrointestinal reactions | Abnormal liver function | Bleeding tendency | Rash | Thrombocytopenia | Blurred vision | Overall  incidence |
| --- | --- | --- | --- | --- | --- | --- | --- |
| Control group 1 (*n*=45) | 0（0.0） | 9（20.0） | 1（2.2） | 0（0.0） | 0（0.0） | 0（0.0） | 10（22.2） |
| Control group 2 (*n*=45) | 0（0.0） | 18（40.0） | 2（4.4） | 0（0.0） | 0（0.0） | 0（0.0） | 20（44.4） |
| Exposed group (*n*=45) | 2（4.4） | 10（22.2） | 4（8.9） | 2（4.4） | 0（0.0） | 0（0.0） | 18（40.0） |
| χ^2^ | 4.060 | 5.436 | 2.109 | 4.060 | - | - | 3.971 |
| P | 0.131 | 0.066 | 0.348 | 0.131 | - | - | 0.137 |

Note: ^*^: *P*<0.05 compared with control group 1, ^#^: *P*<0.05 compared with control group 2.

Control group 1: progesterone combined with enoxaparin sodium; control group 2: aspirin combined with enoxaparin sodium; exposed group: hydroxychloroquine sulphate combined with aspirin and enoxaparin sodium
